# Expanding the Clinical Spectrum of Porokeratosis Ptychotropica

**DOI:** 10.7759/cureus.85727

**Published:** 2025-06-10

**Authors:** Brooke R Bartley, Dylan R Maldonado, Steven Mays, Ronald P Rapini

**Affiliations:** 1 Internal Medicine, Texas Health Presbyterian Hospital, Dallas, USA; 2 Dermatopathology, University of Texas Health Science Center at Houston, Houston, USA; 3 Dermatology, University of Texas Health Science Center at Houston, Houston, USA

**Keywords:** cornoid lamella, diffuse presentation, hyperkeratotic skin lesions, porokeratosis, porokeratosis ptychotropica

## Abstract

Porokeratosis ptychotropica (PP) is a rare variant of porokeratosis, typically presenting as pink to brown hyperkeratotic plaques in the gluteal cleft and buttocks, defined histologically by multiple cornoid lamellae. Diagnosis of PP is challenging due to its clinical overlap with other dermatologic conditions and inconsistent terminology in the literature. This case report describes a 47-year-old woman exhibiting clinical and pathological features of PP but with a more diffuse presentation than previously documented. The case emphasizes the diagnostic complexities of PP, particularly in atypical presentations, and highlights the critical role of histological examination in distinguishing it from other porokeratoses.

## Introduction

Porokeratosis ptychotropica (PP) is a rare variant of porokeratosis, a group of cutaneous disorders characterized by abnormal keratinization and the presence of cornoid lamella on histology. The abnormal keratinization often presents clinically as a distinctive ridge-like border and hyperkeratotic or verrucous papules and plaques [1]. Histologically, cornoid lamella is defined by an invagination of the squamous epithelium forming an epidermal dell, filled with an overlying column of parakeratosis and an underlying loss of the granular cell layer [2]. Five primary clinical variants of porokeratosis have been described: classic porokeratosis of Mibelli, disseminated superficial (actinic) porokeratosis (DSAP), porokeratosis palmaris et plantaris disseminata, linear porokeratosis, and punctate porokeratosis. PP is now increasingly recognized as a distinct subtype within this spectrum.

PP typically presents as pink to brown hyperkeratotic papules and plaques within the gluteal cleft and on the buttocks, with a distinctive histological distribution of multiple cornoid lamellae throughout the lesion [3]. Diagnosis of PP is often challenging due to its overlapping clinical features (such as hyperkeratotic plaques and anatomical distribution) with more common conditions including psoriasis, verruca vulgaris, and dermatophyte infections [4]. Atypical presentations of PP can further complicate diagnosis and delay appropriate treatment, especially when clinical features fall outside the classic distribution or appearance.

As of 2024, fewer than 60 cases of PP have been reported in the literature [5]. Global crude cumulative incidence rates are estimated at only two to four cases per several billion people per year, though this is likely underestimated. The median age of affected patients is approximately 49 years, with a 9:1 male-to-female ratio. PP has been more commonly reported in the United States; however, this may be due to increased recognition, publication bias, or underreporting in other regions rather than a true geographic predilection [5].

This case report describes a 47-year-old woman exhibiting clinical and pathological features of PP but with a more diffuse presentation than previously documented. The case underscores the diagnostic complexities of PP in non-classical presentations and highlights the essential role of histopathology in achieving an accurate diagnosis.

## Case presentation

**Figure 1:**
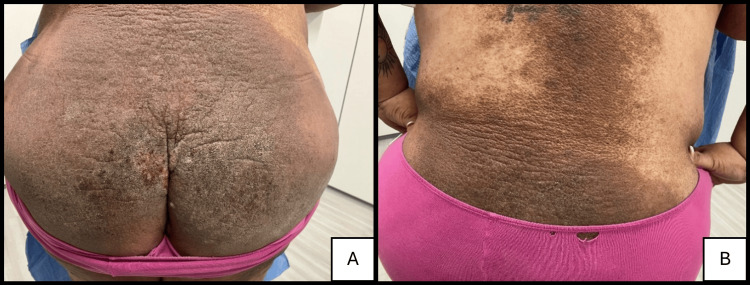
(A) Hyperpigmented, reticulate, and hyperkeratotic plaques on the gluteal cleft with extension to the buttocks. (B) Similar lesions involving the lower and mid-back.

**Figure 2:**
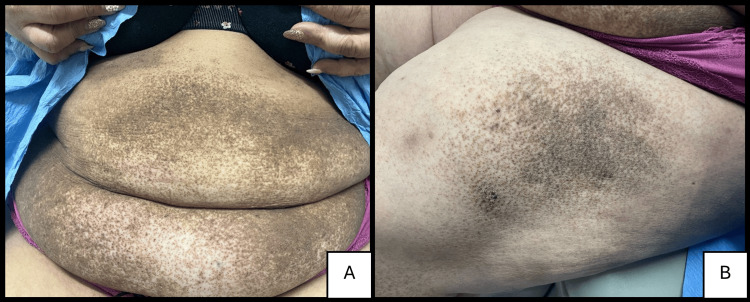
(A) Hyperpigmented, hyperkeratotic plaques on the lower abdomen. (B) Similar lesions on the upper left lateral thigh.

A 47-year-old woman with Fitzpatrick skin type V skin presented with a four-month history of a non-pruritic rash that initially appeared in the gluteal cleft and subsequently spread to the buttocks (Figure 1A), lower back (Figure 1B), abdomen (Figure 2A), lateral thighs (Figure 2B), knees, shins, and upper chest. The lesions were large, hyperpigmented, reticulate, and hyperkeratotic plaques. The patient had a history of widespread psoriasiform dermatitis diagnosed by multiple previous biopsies, treated successfully with mycophenolate. The patient stated that the current eruption did not resemble her previous dermatitis and was not responding to her previous treatment of mycophenolate.

**Figure 3:**
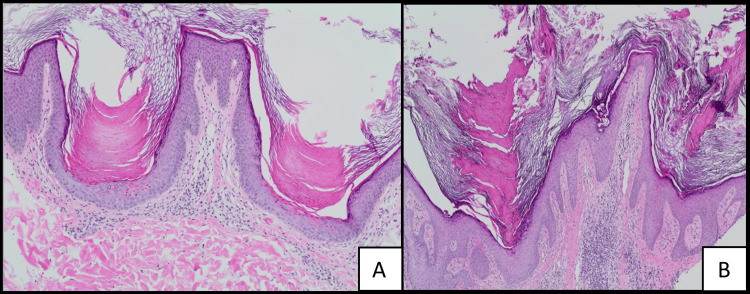
Hematoxylin and eosin stain 20x magnification. (A) Biopsy from the buttock showing multiple cornoid lamellae with dyskeratosis at the bases. (B) Biopsy from the lower back with similar histological findings. Perivascular lymphocytic infiltrate is present in the dermis.

Punch biopsies were obtained from the left buttock and right mid-back. Histopathologic examination of both specimens revealed multiple cornoid lamellae, characterized by columns of parakeratotic cells extending through the stratum corneum, overlying areas of dyskeratosis, and a diminished granular layer. These features were present in both the buttock (Figure 3A) and lower back (Figure 3B) biopsies. A perivascular lymphocytic infiltrate was also observed in the dermis.

The diagnosis of PP was confirmed based on the histopathological findings and supported by a hyperkeratotic patch originating in the gluteal cleft. The patient was started on oral acitretin 10 mg daily, with moderate improvement observed after two months of treatment. Due to the positive initial response, the dosage was increased to 17.5 mg daily. While outcomes after eight months of treatment are still pending, the initial treatment response and confirmed diagnosis represent significant progress in managing this case.

## Discussion

In 1995, Lucker et al. identified and named PP as a distinct form of porokeratosis, due to its specific clinical presentation and unique variation of cornoid lamella observed on histology [6]. The name "ptychotropica" is derived from the Greek words "ptyche" (fold) and "trope" (turning), reflecting the condition's tendency to affect the gluteal cleft [6]. The typical clinical presentation of PP is characterized by symmetrical red to brown hyperkeratotic coalescing papules or plaques in a symmetric, butterfly distribution in the gluteal cleft. While typical PP is often defined by gluteal cleft involvement due to its naming, other studies have shown that PP distribution can expand to other sites including the buttocks, knees, tops of thighs, groin, ankles, chest, and feet [1,7,8]. This case was one of the rare instances where PP extended beyond the gluteal cleft, despite initially appearing in the eponymous site.

This expanded distribution of hyperkeratotic papules and plaques that can be seen in PP is one factor that complicates the diagnosis, leading to frequent misidentification as other dermatoses, including other forms of porokeratosis. For example, genital porokeratosis is a porokeratosis variant that is also reported to present as hyperkeratotic plaques in the genital region. Subtle clinical distinctions can aid in differentiating morphologically similar porokeratosis subtypes. PP is often characterized by a pattern of coalescing plaques originating on the buttocks and expanding to involve genital areas, whereas genitogluteal porokeratosis more commonly presents as discrete papules, which may not exhibit a coalescing pattern [8]. Differential diagnoses can also include psoriasis, condylomas, tinea corporis, ichthyoses, confluent and reticulated papillomatosis, and chronic eczema [4]. Clinically differentiating the hyperkeratotic plaques of PP from other similar conditions is challenging, necessitating biopsy to align clinical and histological findings, as with our patient. This highlights the critical role of clinicopathological correlation in the diagnosis of hyperkeratotic conditions affecting the gluteal region.

Histologically, PP exhibits cornoid lamella, a unifying histologic finding in porokeratoses, though with distinct variation that supports its classification as a unique porokeratosis subtype [3]. Unlike other porokeratosis variants, which typically feature a cornoid lamella at the periphery of the lesion, PP is characterized by multiple cornoid lamellae distributed throughout the lesion [4]. Beneath these lamellae, the granular layer is often absent, accompanied by dyskeratotic cells and focal degeneration of the basal layer. Additionally, some cases of PP have reported amyloid deposits within the papillary dermis [7]. Interestingly, the presence of cornoid lamellae is not pathognomonic for porokeratoses. Cornoid lamella on histology is a reaction pattern that Wade and Ackerman (1980) reported could be seen in many other hyperplastic and neoplastic conditions such as seborrheic keratoses, solar keratoses, squamous cell carcinoma in situ, and basal cell carcinoma [9]. However, the presence of cornoid lamella on histology effectively differentiates PP from most clinically similar diagnoses, such as psoriasis.

The patient’s prior history of psoriasiform dermatitis initially complicated the differential diagnosis. Stone et al. (1999) reported a similar case of PP, where the patient was initially misdiagnosed with psoriasis due to biopsy findings showing psoriasiform acanthosis [3]. However, a subsequent biopsy revealed the presence of cornoid lamellae, leading to the correct diagnosis of PP [3]. In the same way, our patient was initially diagnosed with psoriasiform dermatitis on biopsy. It may be possible that, as reported in the case by Stone et al. (1999), our patient’s initial diagnosis was also incorrect, resulting in a delay in appropriate treatment and permitting diffuse progression of the condition [3]. On the other hand, because the previous psoriasiform dermatitis was treated successfully with mycophenolate, it is also possible that the patient had a non-associated, distinct prior condition before developing PP. The original biopsy diagnosing psoriasiform dermatitis was performed at an outside clinic, and therefore confirmation was not possible.

Given the diagnosis of PP, treatment selection was guided by the extent and severity of lesions. While no standardized treatment protocol exists for PP due to its rarity, broader porokeratosis data suggests that topical retinoids are the most frequently utilized treatment modality (22.0%), followed by oral retinoids (18.6%) and topical corticosteroids (15.2%) [5]. Other treatment options that have been explored for PP include topical and intralesional corticosteroids, cryotherapy, topical 5-fluorouracil, imiquimod, psoralen plus ultraviolet A (PUVA) therapy, and, more recently, simvastatin and abrocitinib [10,11]. Retinoids, both topical and oral, have been favored due to the presumed keratinization defect central to porokeratosis pathogenesis. In this case, the decision to initiate oral acitretin was driven by the diffuse distribution and marked hyperkeratosis, which typically warranted systemic therapy to achieve an adequate response.

## Conclusions

This case underscores the complexity of diagnosing and treating PP, particularly when it presents with an atypical distribution of lesions and overlaps with other dermatologic conditions. Clinicians must be aware of the variable clinical presentations and potential treatment challenges associated with PP. Early recognition is essential to improve outcomes, though patient compliance with follow-up is equally important to ensure long-term success. Dermatologists should maintain a high index of suspicion for PP in recalcitrant hyperkeratotic lesions, especially when initial diagnoses prove unresponsive to standard therapies.
